# Reversal of Doxorubicin-Induced Bone Loss and Mineralization by Supplementation of Resveratrol and MitoTEMPO in the Early Development of *Sparus aurata*

**DOI:** 10.3390/nu14061154

**Published:** 2022-03-09

**Authors:** Sunil Poudel, Marisol Izquierdo, Maria Leonor Cancela, Paulo J. Gavaia

**Affiliations:** 1 Centre of Marine Sciences, University of Algarve, 8005-139 Faro, Portugal; spoudel@ualg.pt (S.P.); lcancela@ualg.pt (M.L.C.); 2 Faculty of Medicine and Biomedical Sciences (FMCB), University of Algarve, 8005-139 Faro, Portugal; 3 PhD Program in Biomedical Sciences, FMCB, University of Algarve, 8005-139 Faro, Portugal; 4 Grupo de Investigación en Acuicultura, Universidad de Las Palmas de Gran Canaria, Taliarte, 35214 Telde, Spain; marisol.izquierdo@ulpgc.es; 5 Algarve Biomedical Center, University of Algarve, 8005-139 Faro, Portugal

**Keywords:** oxidative stress, resveratrol, MitoTEMPO, doxorubicin, bone deformities, mineralization

## Abstract

Doxorubicin is a widely used chemotherapeutic drug known to induce bone loss. The mechanism behind doxorubicin-mediated bone loss is unclear, but oxidative stress has been suggested as a potential cause. Antioxidants that can counteract the toxic effect of doxorubicin on the bone would be helpful for the prevention of secondary osteoporosis. We used resveratrol, a natural antioxidant, and MitoTEMPO, a mitochondria-targeted antioxidant, to counteract doxorubicin-induced bone loss and mineralization on *Sparus aurata* larvae. Doxorubicin supplemented Microdiets increased bone deformities, decreased mineralization, and lipid peroxidation, whereas Resveratrol and MitoTEMPO supplemented microdiets improved mineralization, decreased bone deformities, and reversed the effects of doxorubicin in vivo and in vitro, using osteoblastic VSa13 cells. Partial Least-Squares Discriminant Analysis highlighted differences between groups on the distribution of skeletal anomalies and mineralization of skeleton elements. Calcium and Phosphorus content was negatively affected in the doxorubicin supplemented group. Doxorubicin reduced the mRNA expression of antioxidant genes, including *catalase*, *glutathione peroxidase 1*, *superoxide dismutase 1*, and *hsp90* suggesting that ROS are central for Doxorubicin-induced bone loss. The mRNA expression of antioxidant genes was significantly increased on resveratrol alone or combined treatment. The length of intestinal villi was increased in response to antioxidants and reduced on doxorubicin. Antioxidant supplements effectively prevent bone deformities and mineralization defects, increase antioxidant response and reverse doxorubicin-induced effects on bone anomalies, mineralization, and oxidative stress. A combined treatment of doxorubicin and antioxidants was beneficial in fish larvae and showed the potential for use in preventing Doxorubicin-induced bone impairment.

## 1. Introduction

Oxidative stress is caused by reactive oxygen species (ROS) that are normally generated as by-products of aerobic metabolism during oxidative phosphorylation in mitochondria [1]. The major forms of ROS include the superoxide anions (O_2_^−^), hydrogen peroxide (H_2_O_2_), and free radicals such as hydroxyl radicals (OH). Oxidative stress caused by ROS alters the bone remodeling process, causing an unbalance between osteoclast and osteoblast activity [2]. This can lead to metabolic bone diseases and contribute to the pathogenesis of skeletal system disorders including osteoporosis, characterized by low bone mineral density, decrease in bone mass and density, and deterioration of bone structure, which causes bone fragility and risk of fracture [3,4]. Several clinical studies suggested the involvement of antioxidant and/or pro-oxidant systems in the pathology of bone osteoporosis [5,6,7,8]. Osteoporosis can be classified into various types: primary osteoporosis or idiopathic osteoporosis, age-related osteoporosis, and secondary osteoporosis, where bone loss results from a specific disease or medication [4].

Gilthead seabream (*Sparus aurata*) is a highly valuable commercial species widely cultivated in Europe. Due to its extensive cultivation, the presence of skeletal anomalies is one of the most important bottlenecks in current aquaculture production. Skeletal deformities in farmed teleosts are a persistent problem in aquaculture, posing a high economic burden. Deformed fish are manually sorted out from the production on a regular basis, which results in lower profit [9,10,11]. Previous works showed that 15–50% of seabream juveniles possess severe anomalies detected already at early stages [9,10]. Skeletal anomalies, altered meristic characters, and delayed development can be considered as developmental disturbances, which are the indication of inappropriate nutrition and associated oxidative stress [12,13], inappropriate rearing conditions [9,11], and genetics [11,14]. However, early assessment of skeletal anomalies is often difficult since the presence of slight anomalies is hard to diagnose at early larval stages, but these can later develop into more severe abnormalities affecting the external body shape [14,15,16].

With the advancements in technology, several in vitro systems have been established from mineralizing the tissues of fish. Cells derived from zebrafish calcified tissues can be differentiated into osteoblastic and chondroblastic lineages, as was detected by the analysis of expressing differentiation markers, alkaline phosphatase activity, and extracellular matrix mineralization [17]. Similarly, cells derived from gilthead seabream calcified tissue (ABSa15, VSa13, and VSa16) proved to have excellent mineralization capacity and are currently extensively used for mineralization assays [18,19].

Many factors such as oxidative stress, genetics, epigenetic and nutritional factors have been linked to skeletal abnormalities in teleost fishes under culture conditions. Among the nutritional factors, vitamins, minerals, dietary lipids have also been recognized to influence the development of skeletal malformations [12]. Lipid peroxidation produces toxic compounds such as fatty acid hydroxides, fatty acid hydroperoxides, hydrocarbons, and aldehydes, which damage cellular and subcellular membranes, causing several pathological conditions [12,20]. An endogenous antioxidant defense system is responsible for counteracting this oxidative risk on fish [21]. To maintain the antioxidant defense mechanism, radical scavenging enzymes such as catalase, superoxide dismutase, and glutathione peroxidase scavenge hydrogen peroxide, superoxide, and hydroperoxides, respectively [22]. Therefore, a diet with a high level of polyunsaturated fatty acid, such as docosahexaenoic acid (22:6 *n* − 3) and with an imbalanced content in antioxidant compounds results in oxidative stress, where production of intracellular reactive oxygen species (ROS) increases, negatively affecting proteins, lipids and DNA [20]. 

Resveratrol (RES) is a polyphenolic (3,4′,5-trihydroxystilbene) compound naturally found in a variety of plant foods such as grapes, cranberries, and nuts [23]. RES has anti-inflammatory, estrogenic, antioxidant, and proliferative properties, which can influence bone metabolism [24]. This compound was shown to improve bone mineralization and counteract glucocorticoid-induced bone damage in zebrafish [25]. Similarly, resveratrol also inhibits oxidative stress and prevents mitochondrial damage induced by zinc oxide [26]. During osteoblastic differentiation, RES activated the estrogen-mediated extracellular signal-regulated kinase (ERK) 1/2 signaling pathway regulating differentiation and proliferation [27]. In addition, RES also activated AMP-activated protein kinase (AMPK), which regulates osteoblast differentiation and inhibits bone resorption by acting as a negative regulator of RANKL [28]. 

MitoTEMPO [MT, (2-(2,2,6,6-Tetramethylpiperidin-1-oxyl-4-ylamino)-2-oxoethyl) triphenylphosphonium chloride] is a mitochondria-targeted antioxidant [29]. MT is a superoxide scavenger and a physicochemical compound mimicking superoxide dismutase from mitochondria. It can easily pass through the lipid bilayers and accumulate in the mitochondria [30]. The mitochondria metabolic activity is the primary source of ROS and the principal site of ROS-induced damage. Mitochondrial dysfunction influences osteoblasts through the regulation of mitophagy, apoptosis, and mitochondrial DNA damage. Therefore, improving mitochondrial functions through the application of antioxidants can prevent cytotoxicity and dysfunction in osteoblasts [31]. Mitochondrial ROS are also essential for hypoxic enhancement of osteoclast differentiation [32]. In zebrafish, MT was shown to revert the effect of tafazzin knockdown-induced cellular mitochondrial ROS production and cellular ATP decline, thus suggesting that it can potentially counteract mitochondrial oxidative stress [33].

Doxorubicin (DOX) has been used as an anticancer drug that causes cellular toxicity by inducing a massive accumulation of ROS and reactive nitrogen species [34]. Doxorubicin promotes a direct oxidative injure to DNA [35,36] and generates lipid peroxidation [37,38,39,40,41,42]. By the action of NADPH-dependent reductase, it produces semiquinone free radicals by reducing DOX to DOX semiquinone [43,44]. Under aerobic conditions, redox cycling of adriamycin-derived quinone-semiquinone produces superoxide radicals [45]. On the other hand, adriamycin free radicals are produced by a non-enzymatic mechanism involving iron. Redox reaction of adriamycin with Fe^3+^ produces Fe^2+^-DOX free radical complex, which reduces oxygen to hydrogen peroxide and ROS [44,46,47]. A recent study indicated that pre-menopausal breast cancer patients treated with a combination of DOX/cyclophosphamide exhibited low bone mineral density and significant bone loss [48], suggesting a relationship between DOX treatment and systemic bone loss. Accordingly, exposure to DOX caused a 60% reduction in bone formation in normal rats, suggesting a potential for reduced osteoblast differentiation [49,50]. Zebrafish have been used as a model for the investigation of the molecular mechanism on DOX-induced cardiotoxicity [51] and as a screening tool to study the toxic profile of DOX [52]. When exposed to DOX at different concentrations, zebrafish embryos showed serious developmental toxicity. Zebrafish embryos between 4 to 120 h post-fertilization were exposed to different concentrations of DOX. The higher concentrations showed an acute lethal effect while lower concentrations showed sublethal effects as well as multiple malformations on larvae and embryos. As the concentration of DOX increased, the malformation rate was also increased [53]. Based on these findings, we hypothesized that supplementation of antioxidants could reverse doxorubicin-induced bone loss. 

The aims of this study were to investigate the effects of doxorubicin, resveratrol, and MitoTEMPO on bone development and mineralization and to determine the capacity of antioxidants to counteract the potentially negative effects of doxorubicin on the developing skeleton of *Sparus aurata.*

## 2. Materials and Methods

### 2.1. Micro Diet Preparation

The microdiets with antioxidants and pro-oxidant were prepared by manually mixing squid powder first with water-soluble components, then with fat and lipid-soluble vitamins, and finally, on gelatin dissolved warm water. 150 µM (34 mg/kg) of RES based on Luo et al., 2019 [25] and 10 µM (5 mg/kg) of MT based on Peterman et al. 2015 [54] was used for the preparation of microdiets. While for DOX, non-toxic concentrations were determined by treating of larvae with different concentrations (i.e., 5, 15, 30, and 60 µg/kg) and analyzing the effects on mortality. Lower concentrations (i.e., 5, 15, and 30 µg/mL) did not show toxic effects up to 48 h, while 60 µg/kg of DOX were toxic and showed to increase mortality of larvae at 24 h of exposure. Therefore, 30 µg/kg supplementation of DOX was chosen to use in the trial (Figure S1). RES and DOX were dissolved on polar molecules, whereas MT was dissolved in water. With the help of a grinder (Severin, Suderm, Germany), the dough was compressed, and pellets were prepared. The pellets were dried in an oven at 38 °C for 24 h (Ako, Barcelona, Spain). To obtain different particle sizes (i.e., 500 µm, 250 µm, and 125 µm), the dried pellets were ground (Braun, Kronberg, Germany) and sieved (Filtra, Barcelona, Spain) [20]. The proximate composition of basal diet (control) was Protein—64.46%, Lipid—20.44%, Ash—7.27%, Moisture—9.78% [20,55,56] (Table 1 and Table 2). 

### 2.2. Feeding Trial

Gilthead seabream larvae were obtained from natural spawns from a broodstock kept at the facilities of the Grupo de Investigación en Acuicultura (GIA) (EcoAqua Institute, Las Palmas de Gran Canaria, Spain). Larvae were previously fed with rotifers (*Brachinous plicatilis*) enriched with Ori-Green (Skretting, France) and *Artemia nauplii*. At 30 days after hatching (dah), the larvae were randomly stocked into 18 experimental tanks (200 L light grey color cylinder fiberglass tanks) at a density of 2100 larvae/tank. The larvae were fed with experimental microdiets with added antioxidants and pro-oxidant, either alone or in combination. The antioxidant supplemented microdiets were fed every hour from 8:00 to 20:00, whereas pro-oxidant supplemented microdiet was fed at intervals of 72 h and was continued with a respective combination of control or antioxidant diets. The microdiet combinations were control (CON), resveratrol (RES), MitoTEMPO (MT), doxorubicin + control (DOX), doxorubicin + resveratrol (DOX+RES), doxorubicin + MitoTEMPO (DOX+MT). The combinations treatments were performed by feeding simultaneously with both corresponding diets for each group. After 5 days of feeding, microdiet uptake was checked by microphotographic studies. All 18 tanks were supplied with filtered seawater (37 g/L salinity) at an increasing rate of 0.3–1 L/min along the experimental period. Water entered the tank from the bottom and exited from the top; water quality was tested daily, and no deterioration was observed. Water was continuously aerated (125 mL/min), attaining 6.0–6.2 g/L dissolved O_2_, saturation ranging between 84% and 90%. Water temperature was kept between 20.5 ± 0.5 °C throughout the whole trial.

Growth was determined in larvae at 30 dah by measuring the total length in a stereomicroscope Leica MZ10F (Leica, Wetzlar, Germany) with an attached DFC7000T camera (Leica) and dry weight in a precision analytic balance (larvae were dried at 105 °C until constant weight). Final survival was calculated by individually counting all the live larvae at the beginning and the end of the experiment. 

At the end of the trial, microdiets and larvae samples were washed with distilled water and stored at −80 °C for oxidative stress assessment and mineral content analysis. A group of 100 larvae/tank were collected and fixed with PBS buffered 4% formaldehyde solution for 24 h at 4 °C. Then samples were washed with PBS and preserved in 70% ethanol at room temperature to analyze skeletal anomalies and developmental status. For the analysis of skeletal gene markers and oxidative stress-related gene expression, 30 larvae/tank were kept on RNA later solution and stored at −80 °C. For histology, a group of 15 larvae/tank were fixed with PBS buffered 4% formaldehyde.

### 2.3. Whole-Mount Staining of the Skeleton

To evaluate larvae skeletal anomalies and developmental status, 100 larvae/tank were stained with an acid-free double staining protocol for cartilage and bone adapted from Gavaia et al. [57] and Walker and Kimmel et al. [58]. Whole-mount acid-free double staining was performed using alcian blue 8GX (Sigma-Aldrich, Madrid, Spain) for cartilage and alizarin red S (AR-S) (Sigma-Aldrich) for mineralized tissues [58]. Samples were stained in 0.1% alcian blue 8GX solution (dry weight/volume) with 60 mM MgCl_2_ in 70% ethanol for 3 h and rehydrated with a decreasing concentration of ethanol (96% to 25%) for 2 h. Then, the samples were stained overnight with 0.05% AR-S in 0.5% potassium hydroxide (KOH) (Sigma-Aldrich). Stained samples were cleared with 1% KOH and subsequently transferred through an increasing concentration of glycerol (25% to 100%). Samples were stored in 100% glycerol (Merk Millipore, Billerica, MA, USA) until observation.

### 2.4. Skeletal Anomalies

Specimens stained for cartilage and bone were observed under a stereomicroscope MZ10F (Leica). Skeletal anomalies were classified using a dichotomic indicator, where the letter indicates skeletal element affected and the number indicates typology of the anomaly (Table 3: List of considered anomalies) adapted from Gavaia et al. [59] and Prestinicola et al. [9]. 

The following derived variables were computed for each experimental treatment.

I. Incidence of skeletal anomalies;
II. Deformities charge;
III. The average number of affected areas;
IV. Incidence (%) of skeletal anomalies according to the number of areas affected (severity);
V. Incidence (%) of each skeletal anomaly typology according to the region affected.

### 2.5. Meristic Characters

Meristic character count was carried out on the following elements on 300 larvae/group: total mineralized vertebrae, cranial vertebrae, abdominal/pre-haemal vertebrae, haemal vertebrae, caudal fin vertebrae including urostyle, dorsal fin, and anal fin (pterygiophores and lepidotrichia), and caudal fin (hypurals, epurals, and rays). 

The meristic count was carried out based on the following assumptions:

I. Supernumerary bones were included in the meristic count;
II. Non-completely fused bone elements were counted as distinct elements.

### 2.6. Developmental Stage of the Skeleton

For evaluating the skeletal developmental stage, 300 larvae/group were stained with an acid-free double staining protocol for cartilage and bone adapted from Gavaia et al. [57] and Walker and Kimmel [58], and the skeletal elements were observed under a stereomicroscope MZ10F (Leica). Vertebrae, neural arches and spines, ribs, parhypural, urostyle, hypurals, and caudal-fin rays were examined and were categorized according to the degree of mineralization as mineralized, mineralizing, cartilaginous, and absent. 

### 2.7. Mineral Contents

Samples were dried in the oven at 65 °C for 72 h. After complete drying, samples were weighted, and mineral content was determined by microwave plasma-atomic emission spectrometry (MP-AES 4200, Agilent, Santa Clara, CA, USA). Samples were digested with 65% of nitric acid, and extraction was facilitated with magnetic beads on microwave (Discover SP-D 80, CEM, Matthews, NC, USA) for 9 min. The samples were diluted in 1:10 ratio with milli-Q water. The standards for Calcium and Phosphorus were prepared on 5% nitric acid. The extracted samples were measured by atomic emission spectrometry, and the intensity values of the samples were compared against the standard curve.

### 2.8. Cell Culture and ECM Mineralization Assay

VSa13 cells derived from the vertebra of gilthead seabream (*Sparus aurata*) were maintained as previously described [18,60]. Briefly, cells were cultured in Dulbecco’s modified eagle medium (DMEM) supplemented with 10% fetal bovine serum (FBS), 1% penicillin-streptomycin, 1% fungizone, and 2 mM L-glutamine, and incubated at 33 °C in a 10% CO_2_ humidified atmosphere. Confluent cultures were sub-divided 1:3 every 3–4 days using 0.2% trypsin-EDTA solution. 

ECM mineralization was performed as previously described [18,60]. Confluent VSa13 cells were supplemented with osteogenic medium containing 50 μg/mL of L-ascorbic acid, 10 mM β-glycerophosphate, and 4 mM calcium chloride. The cells were treated with antioxidants (RES and MT) for 21 days with a renewal of the treatment medium twice a week. Treatment with pro-oxidant (DOX) was performed for 3 h twice a week. After 21 days, mineral deposition was examined through AR-S staining as described [61]. AR-S staining was quantified by solubilizing calcium-bound dye in 10% (*w*/*v*) cetylpyridinium chloride, and absorbance was measured by spectrophotometry at 550 nm using a microplate reader [18,60].

### 2.9. RNA Extraction and qPCR

Total RNA was extracted from cell cultures using NZYol Reagent (NZYtech, Lisbon, Portugal). Total RNA (1 µg) was submitted to DNase I treatment (Promega, Madison, WI, USA) for 30 min at 37 °C and reverse-transcribed for 1 h at 37 °C using M-MLV reverse transcriptase (Invitrogen, Waltham, MA, USA), oligo-d(T) universal primer [5′-ACGCGTCGACCTCGAGATCGATG(T)13-3′] and RNaseOUT (Invitrogen). Quantitative real-time PCR (qPCR) assays were performed using the Bio-Rad CFX system (Bio-RAD, Hercules, CA, USA). Gene expression was normalized using β-actin as a housekeeping gene [60], and relative quantification was determined using the ∆∆Ct method [62]. Primers used in this study are listed in Table 4.

### 2.10. Lipid Peroxidation (MDA) Analysis

Lipid peroxidation was determined by the reaction of MDA with thiobarbituric acid substance (TBARS) using Lipid Peroxidation (MDA) Assay Kit from Sigma-Aldrich. Briefly, approximately 20–30 mg of larval tissue per sample was homogenized in 1.5 mL of 20% trichloroacetic acid (*w*/*v*) containing 0.05 mL of 1% BHT in methanol. For this, 2.95 mL of freshly prepared 50 mM thiobarbituric acid solution was added before mixing and heating for 10 min at 100 °C. After cooling, protein precipitates were removed by centrifugation (Sigma-Aldrich 4K15, Taufkirchen, Germany) at 2000× *g*, and the absorbance was measured at 532 nm in a Evolution 300 spectrophotometer (Thermo Scientific, Loughborough, UK). The absorbance was normalized against a blank at the same wavelength and was compared with the MDA standard curve. The concentration of TBA-malondialdehyde (MDA) was expressed as nmol MDA per mg of tissue [63]. 

### 2.11. Histology

Histological procedures were performed as previously described in Cardif et al. [64]. The larvae were decalcified with 10% EDTA and 1% PFA prior to paraffin inclusion. Sections were prepared at 5 μm using a Microm HM 315 rotary microtome (Microm International GmbH, Walldorf, Germany) and stained using Harris hematoxylin and eosin as described by Fischer et al. [65]. Blind evaluation of histological preparation was performed, analyzing intestinal villi length [66,67]. Images were acquired with wave image software were processed, and length was measured using ImageJ1.53c software.

### 2.12. Statistical Analysis

Data obtained from the analysis were plotted on an Excel sheet. For the skeletal anomalies, the data were coded according to the typology of the anomaly (Table 3: List of considered anomalies) according to the affected regions, and relative frequency of the anomalies, incidence of anomalies, deformities charge, the relative frequency of affected areas was calculated and analyzed. For the numeric value such as the number of mineralized vertebrae, length, weight, and survival were directly calculated. The data were coded for the analysis of developmental stage as mineralized, mineralizing, cartilaginous, and absent, the numeric value was entered, and finally, the cumulative percentage was calculated. For the meristic count, the absolute true value was entered, and mean, median, and range (maximum and minimum value) were calculated. Partial Least-Squares Discriminant Analysis (PLS-DA), the performance is measured using prediction accuracy or group separation distance using the “B/W ratio” as suggested by Bijlsma et al. [68]. Univariate and multivariate analyses were performed using the MetaboAnalystR 3.0 R package and MetaboAnalyst 5.0 [69,70]. Statistical analysis was performed using IBM SPSS 16 and Graphpad prism 8. Results are expressed as mean ± SEM. Levene’s test was performed for the homogeneity of variance. Significances were evaluated by student’s *t*-test, one-way and two-way Anova. Difference in value *p* ≤ 0.05 was considered significant (ns—*p* > 0.05; *—*p* ≤ 0.05; **—*p* ≤ 0.01; ***—*p* ≤ 0.001; ****—*p* ≤ 0.0001). 

## 3. Results

### 3.1. Doxorubicin Affects Growth and Survival

The toxic dose of DOX was determined by exposing gilthead seabream larvae to different concentrations of DOX for up to 48 h. 60 µg/mL of DOX were toxic for the larvae already at 24 h of exposure, while lower concentrations (i.e., 5, 15, and 30 µg/mL) did not show toxic effects up to 48 h. Therefore, 30 µg/mL concentration of DOX was chosen for the trial (Figure S1). The prepared microdiets showed no toxic effects on larvae (Figure S1). Temperature, oxygen, and O_2_ saturation were stable throughout the trial period (Table S1).

**Figure 1:**
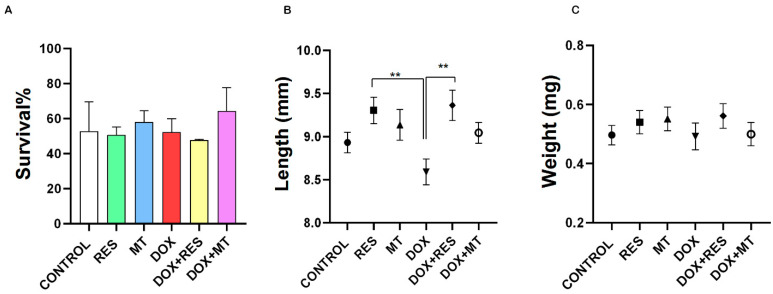
Survival and growth parameters of gilthead seabream larvae at the end of the trial. Survival of seabream (**A**), total length (**B**), dry weight (**C**). One-way ANOVA, Tukey’s multiple comparisons test, **—*p* ≤ 0.01. Acronyms: resveratrol (RES), doxorubicin (DOX), MitoTEMPO (MT), doxorubicin + resveratrol (DOX+RES) and doxorubicin + MitoTEMPO (DOX+MT).

After 15 days of feeding trial (at 45 dah), the overall survival of the larvae was not significantly different between the groups (Figure 1A). The treatment with DOX supplemented diet significantly reduced the total length, but this effect was significantly reversed by co-treatment with RES (Figure 1B). No significant differences were observed in other groups. No significant differences between the groups were found on dry weight (Figure 1C). 

### 3.2. Histological Changes on Antioxidant and Pro-Oxidants Supplemented Groups

**Figure 2:**
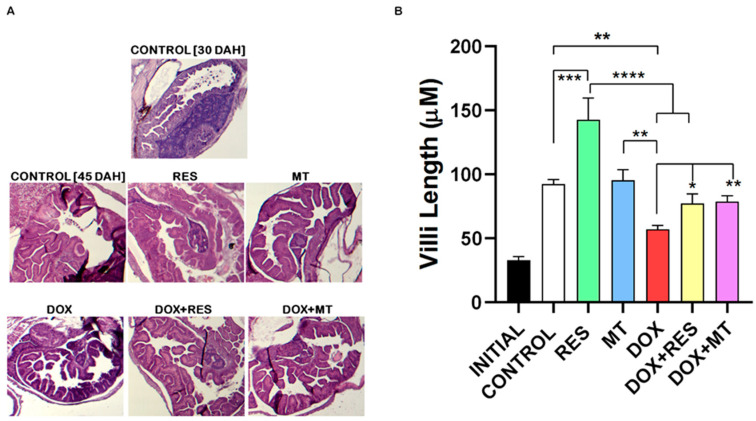
Histology of gut of gilthead seabream supplemented with antioxidants and pro-oxidants microdiets at 45 DAH (days after hatched). Stained with hematoxylin and eosin (10×) initial-initial larvae of 20 dah (**A**). Length of villus measured on ImageJ (**B**). One-way ANOVA, Tukey’s multiple comparisons test,*—*p* ≤ 0.05, **—*p* ≤ 0.01, ***—*p* ≤ 0.001, and ****—*p* ≤ 0.0001. Acronyms: resveratrol (RES), doxorubicin (DOX), MitoTEMPO (MT), doxorubicin + resveratrol (DOX+RES) and doxorubicin + MitoTEMPO (DOX+MT).

Histological sections stained with hematoxylin and eosin (Figure 2A) revealed that DOX supplementation significantly reduced the length of villi as compared to control and antioxidants (RES and MT). In contrast, RES supplementation increased the length of the villi as compared to the control (Figure 2B). The length of the villi on co-treatment of DOX with antioxidants (RES and MT) was not significantly different from the control group but was significantly different from DOX treated group. Therefore, supplementation with both antioxidants significantly protected against the DOX-induced impact on the intestinal mucosa. 

### 3.3. Antioxidants Prevented the Development of Skeletal Deformities

**Figure 3:**
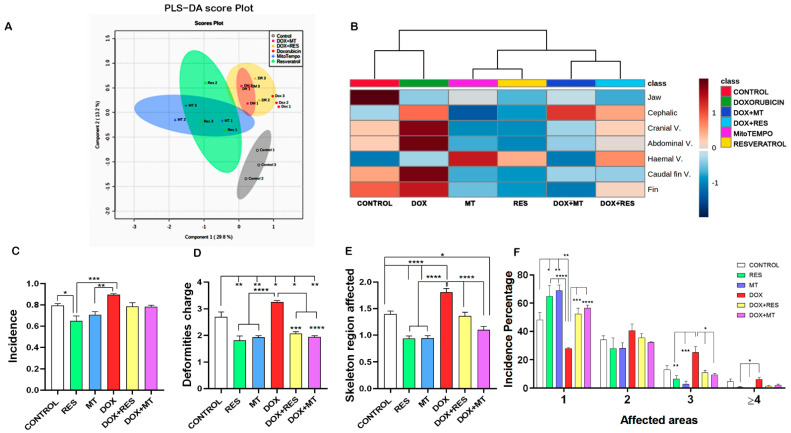
Incidence of skeletal deformities. Partial Least-Squares Discriminant Analysis (PLS-DA score) between the antioxidants and pro-oxidants groups (**A**), heatmap of distribution of skeletal deformities (**B**), incidence of Deformities (**C**), deformities charge (**D**), number of affected areas (**E**), severity of malformation (number of affected areas) (**F**). One-way ANOVA, Tukey’s multiple comparisons test, *—*p* ≤ 0.05, **—*p* ≤ 0.01, ***—*p* ≤ 0.001, and ****—*p* ≤ 0.0001. Acronyms: resveratrol (RES), doxorubicin (DOX), MitoTEMPO (MT), doxorubicin + resveratrol (DOX+RES) and doxorubicin + MitoTEMPO (DOX+MT).

The similarities and discriminant analysis on the distribution of skeletal deformities between the treatment groups were done using Partial Least-Squares Discriminant Analysis (PLS-DA score). The PLS-DA cluster analysis showed three distinct clusters, with the control groups being completely isolated, another cluster with antioxidants (RES and MT), and the third cluster with DOX alone or in combination (Figure 3A). The heatmap shows the distribution of skeletal deformities between the treatment group. The control and DOX treated groups showed a high number of skeletal deformities, whereas the RES treatment group showed a smaller number of skeletal deformities in specific regions. The combination of DOX with either RES or MT prevented DOX-induced skeletal deformities (Figure 3B). 

The incidence of skeletal anomalies was significantly decreased on RES supplemented group as compared to other groups, while the incidence of skeletal anomalies was significantly higher on DOX supplemented group as compared to RES and MT groups (Figure 3C). Similarly, deformities charge and affected areas were significantly increased in the presence of DOX supplementation. But those effects were significantly rescued when DOX was combined with antioxidants (RES and MT) (Figure 3D,E). Thus, supplementation of antioxidants was significantly reduced, while pro-oxidant (DOX) significantly increased the incidence of skeletal deformities, deformities charge, and severity. When analyzing the number of areas affected, it is clearly shown that DOX has induced higher numbers of multiple skeletal deformities. Upon antioxidants treatment, alone or in combination, a significantly higher number of fish presented a less severe phenotype (a smaller number of deformities/affected areas) as compared to DOX treatment (Figure 3F). The larvae supplemented with DOX presented a relatively higher severity of malformations affecting three regions as compared to the groups supplemented with RES and MT. However, DOX supplemented with a combination of antioxidants RES and MT significantly reduced these negative effects. A similar effect was also observed on the severity of malformations affecting four and more regions, with the DOX group showing a significantly higher number of fish with malformations as compared to antioxidants RES and MT (Figure 3F). 

**Figure 4:**
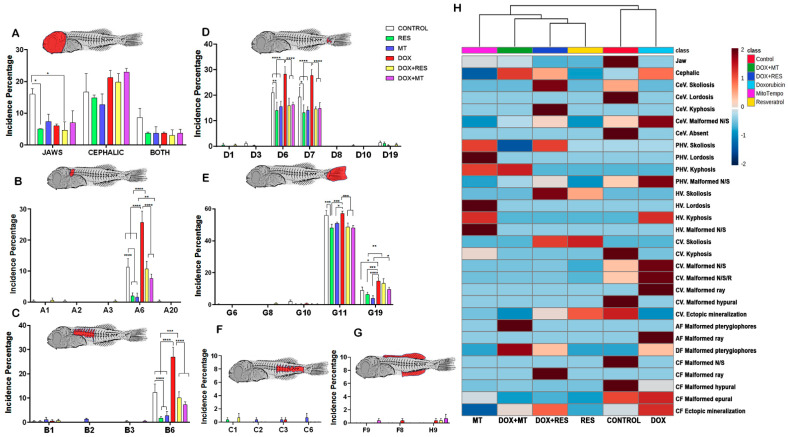
Distribution of skeletal deformities. Incidence of deformities in between the antioxidant and pro-oxidant supplemented groups. Head region (**A**), cephalic region (**B**), prehaemal region (**C**), caudal region (**D**), caudal fin (**E**), haemal or pre-caudal region (**F**), anal fin (**F**), dorsal fin (**G**), and heat map of the specific deformities between the antioxidant and pro-oxidant supplemented groups (**H**). Two-way ANOVA, Tukey’s multiple comparisons test, *—*p* ≤ 0.05, **—*p* ≤ 0.01, ***—*p* ≤ 0.001, and ****—*p* ≤ 0.0001. Acronyms: resveratrol (RES), doxorubicin (DOX), MitoTEMPO (MT), doxorubicin + resveratrol (DOX+RES), doxorubicin + MitoTEMPO (DOX+MT), Cephalic vertebrae (CeV), Pre-haemal vertebrae (PHV), Haemal vertebrae (HV), Caudal vertebrae (CV), Anal fin (AF), Dorsal fin (DF), and Caudal fin (CF).

The incidence of specific skeletal anomalies was analyzed in the different groups (Figure 4). The incidence of jaw deformities showed a significantly lower level on RES supplemented groups as compared to Control. A higher number of cephalic deformities was observed on DOX supplemented groups compared to RES and MT supplemented groups (Figure 4A). DOX supplemented group showed a substantially higher incidence of deformities on cephalic neural arches and/or spines (Figure 4B, A6) as compared to Control, RES, and MT. The incidence of skeletal malformations on these structures was significantly decreased on antioxidant supplemented groups as compared to controls, and the number of deformities significantly reversed when DOX was combined with antioxidants (RES and MT). Similar to the observed in the cephalic region, larvae supplemented with DOX showed a significantly higher incidence of malformations on pre-haemal neural arches and/or spines (B6) as compared to Control and to the groups supplemented with antioxidants RES and MT (Figure 4C). The incidence of skeletal malformations of the neural arches and/or spines on the pre-haemal region was also significantly decreased on antioxidant supplemented groups as compared to controls. The higher incidence of deformities recorded in the caudal region was on the neural arches and/or spines (D6) and on haemal arches and/or spines (D7). The incidence of malformations on D6 has significantly reduced upon RES and MT supplementation as compared to controls, while it was significantly higher upon supplementation with DOX as compared to controls RES and MT (Figure 4D). However, the combination of DOX with antioxidants, RES, and MT substantially reversed the effect of DOX alone. The incidence of malformed epurals (deformed, absent, fused, supernumerary) (G11) and ectopic mineralization (G19) was higher as compared to other deformities in this region. The RES treatment significantly decreased the incidence of malformed epurals as compared to Control. The incidence of malformed epurals was significantly increased by DOX supplementation as compared to RES and MT, and the supplementation with antioxidants significantly reversed that effect (Figure 4E). Ectopic mineralized structures (G19) on the caudal fin region were significantly higher in the DOX group as compared to controls, RES, and MT (Figure 4E). A comparatively low incidence of skeletal malformations was observed on the haemal vertebrae region (Figure 4F) and on the anal and dorsal fins (Figure 4G). The heat map showed an overall distribution of skeletal anomalies between the groups, with two distinct clusters, with control and DOX groups being different from the other groups (Figure 4H). 

The analysis of the results on skeletal deformities clearly demonstrates that the incidence of malformations was higher in the DOX group than in the RES and MT groups. Similarly, when the antioxidants RES and MT were combined with the pro-oxidant, the DOX-induced negative effects on the incidence of particular malformations were significantly reversed (Figure 3 and Figure 4). 

### 3.4. Antioxidants Prevent Dox-Induced Delays in Mineralization and Development

**Figure 5:**
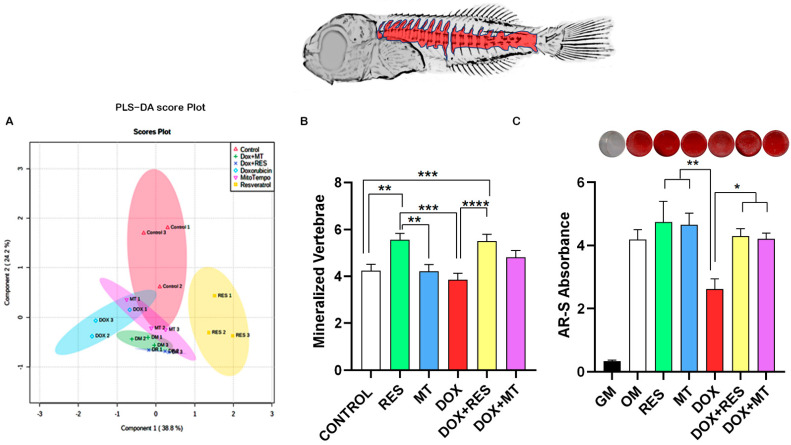
Mineralization of the skeletal elements. Partial Least-Squares Discriminant Analysis (PLS-DA score) between the antioxidants and pro-oxidants groups on the mineralization of the skeleton elements (**A**), Mineralized vertebrae of seabream fed with Microdiets (**B**), and Extracellular matrix mineralization assay of Vsa13 cells (**C**). One-way ANOVA, Tukey’s multiple comparisons test, *—*p* ≤ 0.05, **—*p* ≤ 0.01, ***—*p* ≤ 0.001, and ****—*p* ≤ 0.0001. Acronyms: Alizarin red-S (AR-S), Growth medium (GM), Osteogenic medium (OM), resveratrol (RES), doxorubicin (DOX), MitoTEMPO (MT), doxorubicin + resveratrol (DOX+RES), and doxorubicin + MitoTEMPO (DOX+MT).

To illustrate the differences in skeleton mineralization, we performed Partial Least-Squares Discriminant Analysis between the treatment groups on the mineralization pattern of skeletal elements on whole-mount stained larvae. The cluster analysis revealed that mineralization of the skeleton elements between control, antioxidant, pro-oxidant, and antioxidants plus pro-oxidant groups were distinctly different from one another (Figure 5A). Acid-free double staining using alcian blue and alizarin red showed that RES supplementation significantly increased mineralization of vertebrae as compared to Control. Whereas MT supplementation did not show any difference in the mineralization of vertebrae and DOX supplementation significantly decreased the mineralization of vertebrae as compared to RES. Interestingly, the combined treatment with DOX and RES significantly reversed the effect of DOX on the mineralization of vertebrae, providing levels comparable to DOX treatment alone (Figure 5B). The mineralization pattern of skeletal elements between the antioxidants and pro-oxidants supplemented groups observed in the larvae was confirmed with an in vitro extracellular matrix mineralization assay using VSa13 cells. AR-S staining was performed to investigate extracellular matrix mineralization. The exposure of Vsa13 cells to DOX significantly impaired the mineralization capacity of the cells as compared to treatments with RES and MT. In contrast, upon treatment in combination with RES or MT, the effect of DOX was significantly reversed (Figure 5C), as confirmed by the increase in mineralization of osteoblastic cells. 

**Figure 6:**
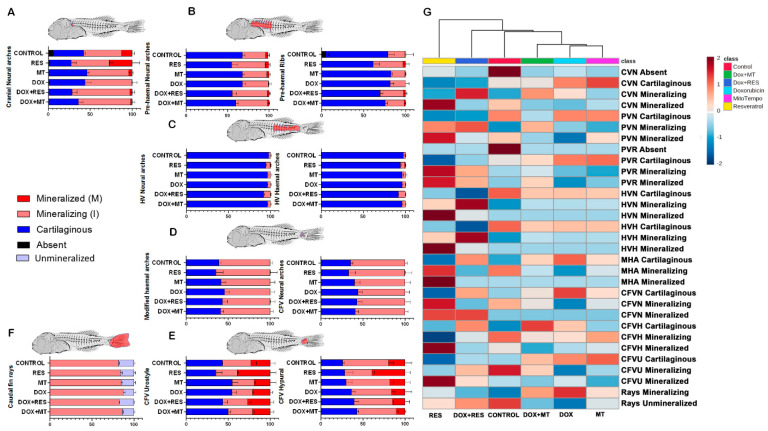
Developmental status and mineralization patterns of gilthead seabream fed different microdiets. Percentage of mineralized, mineralizing, cartilaginous, unmineralized and absent; cranial vertebrae neural arch (**A**), prehaemal vertebrae neural arches and ribs (**B**), haemal vertebrae neural (**C**), modified haemal arch and caudal vertebrae neural arches (**D**), caudal fin vertebrae (CFV), urostyle and hypural (**E**), and caudal fin rays (**F**). Heat map of mineralization pattern of skeleton element between the antioxidant and pro-oxidant supplemented groups (**G**). Graph: percentage +/− SEM. Acronyms: resveratrol (RES), doxorubicin (DOX), MitoTEMPO (MT), doxorubicin + resveratrol (DOX+RES) and Doxorubicin + MitoTEMPO (DOX+MT), Cephalic vertebrae neural arches (CVN), Pre-haemal vertebrae neural arches (PVN), Pre-haemal vertebrae ribs (PVR), Haemal vertebrae neural arches (HVN), Haemal vertebrae haemal arches (HVH), Caudal vertebrae Modified haemal arches (MHA), Caudal fin vertebrae neural arches (CFVN), Caudal fin vertebrae hypural (CFVH), Caudal fin vertebrae urostyle (CFVU), and Caudal fin rays (Rays).

The RES supplementation increased mineralization in neural arches of cranial vertebrae (27% mineralized and 45% mineralizing) as compared to other groups (Figure 6A). The MT-treated group showed a higher percentage of cartilaginous (46%) and mineralizing (49%) structures, while only 5% were already mineralized. The DOX supplemented group showed delayed mineralization with a large portion of the structures still cartilaginous (43%) or mineralizing 55% (Figure 6A). Similarly, in pre-haemal neural arches and ribs, RES supplementation was shown to increase the percentage of mineralizing (RES-41%, MT-30%) and mineralized (RES-5%, MT-3%) structures, while in the DOX treatment, no mineralized arches were observed (Figure 6B). Haemal vertebrae, neural arches, and spines were still under formation on 45 dah seabream larvae. All groups showed 90% and more cartilaginous neural arches and spines. (Figure 6C). In caudal fin vertebrae, the groups treated with antioxidants (RES and MT) showed an increased number of mineralizing neural arches and modified haemal arches compared to the DOX supplementation group, where the number of cartilaginous (45%) structures was higher (Figure 6D). RES and MT increased the mineralization, as well as mineralizing state of urostyle and hypurals. DOX supplementation increased cartilaginous elements, whereas in combination with antioxidants, the percentage of mineralizing and mineralized urostyle was increased compared to DOX alone (Figure 6E). Antioxidant supplementation showed a slight increment in mineralizing caudal-fin rays. The DOX supplementation did not show any difference in mineralizing rays percentage (Figure 6F). The heatmap shows the pattern of mineralization of the skeletal elements between the treatment groups. The control and DOX treated groups showed a lower intensity of mineralization, whereas the RES treatment groups showed a high intensity of mineralization of skeletal elements. The combination of DOX with RES or with MT partially prevented DOX-induced reduction of mineralization (Figure 6G). 

### 3.5. Antioxidant and Pro-Oxidants Alter the Meristic Characters

The meristic character counts are shown in Table 5, with a comparison of observed mean, median, and range values between the groups supplemented with antioxidant, pro-oxidant, and the combination of both. The number of mineralized vertebrae was different among the groups, with a higher number observed in the RES supplemented group and a decreased number in DOX supplemented group (Table 5 and Figure 5A,B). The mean number of mineralized cranial vertebrae was significantly different on the RES group compared to DOX and control groups (one-way ANOVA *p* < 0.0001). The number of pre-haemal vertebrae and the haemal vertebrae showed no differences between the groups. In the caudal region, the number of epurals was increased in DOX supplemented group, whereas in other groups, there were no differences. Significant differences were observed on ectopic cartilage in hypuralia between the groups as well as in the number of caudal-fin rays. In the dorsal fin, both the number (mean and median) of cartilaginous pterygiophores and of unmineralized lepidotrichia were higher on the DOX group as compared to other groups. The number (mean and median) of cartilaginous pterygiophores on the anal fin, as well as the number of unmineralized lepidotrichia, were also higher on DOX as compared to other groups.

### 3.6. Doxorubicin Affects Minerals Content

**Figure 7:**
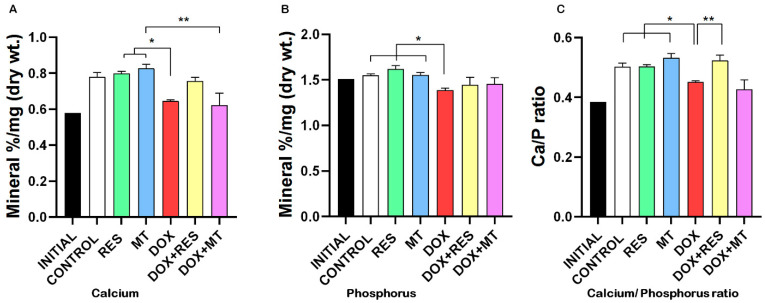
Mineral analysis of gilthead seabream fed with microdiets. Calcium (**A**), phosphorus (**B**), calcium/phosphorus ratio (**C**). One-way ANOVA, Tukey’s multiple comparisons test, *—*p* ≤ 0.05, **—*p* ≤ 0.01. Acronyms: resveratrol (RES), doxorubicin (DOX), MitoTEMPO (MT), doxorubicin + resveratrol (DOX+RES), and doxorubicin + MitoTEMPO (DOX+MT).

Although the mineral formulation of the microdiets was similar (Table 1 and Table 2), analysis of mineral content showed that the DOX group had significantly lower calcium and phosphorus contents compared to RES and MT groups (Figure 7A,B). The calcium/phosphorus ratio was also significantly reduced in the group supplemented with DOX as compared to other groups except for DOX+MT (Figure 7C). Similarly, potassium content was significantly reduced in the group supplemented with DOX as compared to the groups supplemented with RES and MT alone or in combination with DOX (Figure S2). Sodium, magnesium, and iron contents were also decreased in the groups supplemented with DOX alone or in combination with antioxidants (Figure S2). 

### 3.7. Doxorubicin-Induced Oxidative Stress Was Reversed by Antioxidants

**Figure 8:**
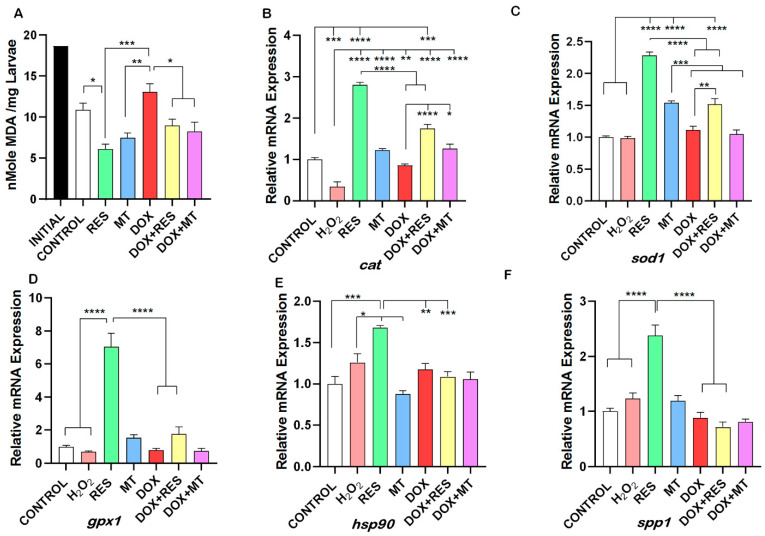
Oxidative stress and antioxidant status on antioxidant and pro-oxidant treatment. Lipid peroxidation of the gilthead seabream larvae feed with antioxidant and pro-oxidant diets, initial- initial larvae of 20 days (**A**). mRNA expression of expression of antioxidants genes on VSa13 cells treated with Antioxidants and Pro-oxidants (Resveratrol, MitoTEMPO, Hydrogen peroxide and Doxorubicin); *Catalase* (*cat*) (**B**), *superoxide dismutase 1* (*sod1*) (**C**), *glutathione peroxidase 1* (*gpx1*) (**D**), *heat shock protein 90 kDA alpha 1* (*hsp90*) (**E**) and *osteopontin* (*spp1*) (**F**). One-way ANOVA, Tukey’s multiple comparisons test, *—*p* ≤ 0.05, **—*p* ≤ 0.01, ***—*p* ≤ 0.001, and ****—*p* ≤ 0.0001. Acronyms: Resveratrol (RES), Doxorubicin (DOX), MitoTEMPO (MT), doxorubicin + resveratrol (DOX+RES), doxorubicin + MitoTEMPO (DOX+MT).

An increase in free radicals causes overproduction of Malondialdehyde (MDA), a common marker of oxidative stress and antioxidant status. The degree of lipid peroxidation was significantly higher in the DOX supplemented group as compared to the RES and MT groups. No significant difference was seen between DOX and the control group. RES supplementation significantly reduced the lipid peroxidation as compared to the control group. Similarly, in the groups combining DOX with antioxidants, the MDA level was significantly reduced as compared to DOX alone (Figure 8A). In the DOX treated groups, a significant reduction was observed in mRNAs transcribed from antioxidant genes, including *catalase*-*cat* (Figure 8B), *superoxide dismutase 1—sod1* (Figure 8C), *glutathione peroxidase 1*—gpx1 (Figure 8D), and *heat shock protein 90—hsp90* (Figure 8E) as compared to RES. *Osteopontin* (*spp1*), a gene responsible for the mineralization and highly expressed by mature osteoblasts was significantly upregulated upon RES treatment, whereas *spp1* expression was significantly reduced on DOX treated group as compared to RES (Figure 8F).

## 4. Discussion

Doxorubicin is one of the main anticancer drugs, causing high toxicity characterized by massive accumulation of ROS [71] and reactive nitrogen species as its central working mechanisms [34,72,73]. As a result, DOX promotes direct oxidative damage to DNA [35,36] and increases lipid peroxidation [37,38,39,40,41,42]. DOX-treated patients exhibit significant bone loss [48], and a reduction by 60% of bone mass was also observed in normal rats treated with DOX [49,50]. 

The developmental toxicity associated with DOX has been previously studied in various models, including rats [74], dogs [75], and zebrafish [51,52,53]. In zebrafish, DOX induces developmental toxicity, with higher concentrations (≥25 mg/L) causing acute lethal effects and lower concentrations (≤0.1 mg/L) showing sublethal effects with multiple malformations [53]. In this study, we aimed to counteract the doxorubicin-induced bone loss by antioxidant supplementation. Our data shows that DOX supplementation (30 mg/kg) promoted a delay in growth when compared to RES supplementation, as indicated in total length; however, the weight of the larvae did not show any difference. Reduction of growth is considered a marker for chronic stress in teleost fish [76], and antioxidant supplementation has been shown to improve growth on rainbow trout and southern flounder and rescue the glucocorticoid-induced negative effects on growth in zebrafish [25,77,78]. Similarly, MT had been shown to improve development in re-implanted porcine embryos [79]. 

The dietary nutrients are metabolized and absorbed in the jejunum with intestinal villi increasing the surface area of absorption [80]. Up to 75% of RES is absorbed by passive diffusion after oral administration, accumulating in several organs, such as the stomach, intestines, and liver, where it is significantly absorbed and metabolized [81]. Previously, it was observed that DOX administration increased apoptosis of the jejunal epithelium [82], demonstrated by severe intestinal damage, reduction of villus length, and an increased influx of leukocytes [83]. The pharmacokinetics examination of plasma DOX after oral administration revealed that the maximum concentration (Cmax) was 0.2062 l/mL, and the maximum time (Tmax) was 2 h [84]. Therefore, in the present study, we examined the effect of DOX on intestinal villi and investigated if antioxidants were able to protect them against pro-oxidant-induced oxidative stress. Comparable to the results reported by Zhou et al. [85], the villi length was increased in response to antioxidants treatment. The absorption and pharmacokinetics of these compounds can be pointed to as factors partially responsible for the observed effects. The negative impact of DOX on intestinal villi may thus be the reason for the delayed growth of larvae fed with DOX supplementation. 

Skeletal anomalies are detectable at very early stages and can develop into sub-lethal anomalies in subsequent life stages [11]. They are more prone to occur in animals having fast growth rates [86,87] since 90% of the bone’s organic content is represented by collagen, responsible for maintaining stability and mechanical function [87]. Ascorbic acid deficiency in fish has been shown to decrease bone collagen content and increase the incidence of skeletal anomalies [88]. Skeletal anomalies established during early stages were characterized by disorganized connective tissue, abnormal mineralization, and muscle bundles [89,90]. The anomalies in the skull, jaw, pre-maxillary, glossopharyngeal and opercular plate affect the efficient nourishment of the larvae due to their inability to feed, which results in slower growth and weaker larvae [91,92]. Our results showed that antioxidants significantly decreased the incidence of jaw anomalies resulting in better nourished and stronger larvae. In the current study, the caudal (vertebrae and fin) region was found to be the most strongly affected on all groups, confirming what had previously been reported by some authors for this species [9,91,92,93]. Anomalies affecting the vertebral arches and ribs are considered insignificant since they have no impact on the external shape [11]. However, the presence of these anomalies is a sign of altered osteogenic processes, and since neural arches protect the spinal cord and provide an entry point for dorsal musculature, severe anomalies in neural arches and spines will affect the overall performance of the larvae [10,11]. The haemal arches protect the arteria and venae caudalis, therefore severe anomalies in these structures could interfere with normal blood flow resulting in chondrogenesis influenced by low oxygen levels [94]. Santamaria et al. [89] observed gilthead seabream larvae with lordosis at early stages, before the vertebrae differentiate, due to the disorganized mineralization process. In European seabass and red seabream, lordosis are associated with failure to inflate the swim bladder, resulting in increased swimming activity which can overall affect the development of the larvae [95]. Due to high swimming activity, the larvae overall energy expenditure is increased, and consequently its demand for food intake [96]. The caudal vertebrae and the caudal fin were the most affected region in this study with a higher incidence of skeletal anomalies, as previously reported for *S. aurata* [9,93,97]. The incidence of skeletal anomalies on caudal vertebrae and caudal fine were found to be significantly higher upon DOX supplementation, whereas when combined with antioxidants (RES and MT), the incidence was significantly reduced compared to DOX alone. Divanach et al. [98] and Koumoundouros et al. [97] have indicated that incidence of the caudal deformities can induce secondary vertebral deformities or reduce the biological performance (growth, conversion index) of the fish due to their effect on swimming efficiency. Accordingly, we found that vertebral arches/ribs and neural arches were significantly affected upon DOX supplementation, which may be the result of altered osteogenic process and mineralization. These effects were significantly rescued with the co-supplementation of antioxidants (RES and MT), suggesting that it can effectively prevent pro-oxidant-induced bone deformities.

Studies by Boglione et al. [10,11] showed that skeleton anomalies detected at early ontogenetic stages (poorly or not differentiated skeletal tissue) could develop into sub-lethal skeletal anomalies at later stages. In addition, even if some vertebrae and related anomalies can develop at an older age, many skeletal anomalies arise during chondrogenic and osteogenic differentiation during early larval stages [10,11,99,100]. The mineralization pattern of the skeletal elements was analyzed in this study and found to be altered between the antioxidant and pro-oxidant supplemented groups in vivo, a result also confirmed with in vitro experiments. Mineralization and development of the axial skeleton of seabream larvae are simultaneous to posterior notochord dorsal flexion and sequentially posterior to the hypural complex and urostyle [15]. RES supplementation significantly increased the mineralization of the skeletal elements in the larvae, which can be the result of increased osteoblast differentiation and mineralization, as confirmed by the VSa13 mineralization assay. Osteopontin (*Spp1*) plays an important role in the regulation of biomineralization [101,102]. Preosteoblastic cells express *Spp1* mRNA at early bone differentiation stages, while the highest expression of *spp1* is observed in mature osteoblasts [103]. The expression of *spp1* mRNA is directly proportional to the ALP activity and calcium deposition level [104], and *Spp1* is upregulated by growth, differentiation factors, and by mechanical stress, which promotes bone formation [20,103,105]. Down regulation of *spp1* is associated with low mineralization in mechanically stimulated mice [105]. VSa13 cells showed a decrease in the expression of *spp1* upon DOX treatment compared to RES. These data on VSa13 cells EC mineralization assay further strengthen the in vivo mineralization of seabream vertebrae on DOX-induced bone loss.

The diversity of mineralization patterns during the development of skeletal elements in teleosts indicates the complexity of the mechanisms involved [15]. Previously, Prestinicola et al. [9] and Russo et al. [106] have shown that the variability on the meristic counts is directly related to the occurrence of skeletal anomalies. In this study, we observed the alteration of meristic characters between the antioxidant and pro-oxidant treated groups of seabream larvae. Furthermore, antioxidant supplementation increased mineralization and reversed the pro-oxidant induced negative effects on mineralization, both in vitro and in vivo, suggesting that RES [25,26,107] and MT [108] supplementation would be beneficial, not only for enhancing bone development and mineralization but also for the overall development of larvae. 

In higher vertebrates, the skeleton serves as the main reservoir for calcium and phosphate [90]. Skeletal system development and vertebrae stability are related to the mineral content. Fish absorb various elements from water, including calcium; therefore, calcium deficiency is uncommon in fish. In contrast, food is the main source of phosphorus for fish, with low phosphorus intake resulting in reduced bone mineralization, skeletal abnormalities, and reduced growth [109,110]. Several in vitro and in vivo studies have shown that DOX reduces mitochondrial calcium uploading capacity [111,112,113,114]. Indeed, the increased induction of mitochondrial permeability transition pore resulted in a loss of calcium loading capacity [112,115]. Antioxidants are able to protect against DOX-induced mitochondrial toxicity [111]. Our results showed that DOX significantly reduced the Ca/P ratio as compared to control and antioxidant supplementation, whereas combined treatment with RES significantly rescued the Ca/P ratio. In addition, treatment with DOX decreased the calcium and phosphorus content of the larvae, suggesting its interference with mineral metabolism. Similarly, a decrease in the levels of potassium has been shown to increase bone resorption activity [116]. In our study, DOX significantly decreased potassium content as compared to antioxidants supplementation (Figure S2), suggesting that DOX might increase osteoclast differentiation. 

One of the objectives of this study was to illustrate the mechanism of DOX-induced bone loss. Our data indicate that supplementation of DOX reduced the expression of antioxidant genes, including *cat*, *gpx1*, *sod1*, and *hsp90* [107,117], which was significantly increased upon RES supplementation, alone or in combination with DOX. These results suggest that DOX-induced bone loss is the result of oxidative stress [72], which was rescued upon RES supplementation. Similarly, Meng et al. [118] also observed the improvement of antioxidant status regulating antioxidant genes when RES was supplemented through diet in sows and piglets [118]. The DOX treatment induced an increase in lipid peroxidase, a decrease in antioxidant gene expression, and in osteoblastic-associated mineralization. The ability of RES [26,107] and MT [108,119] to counteract those effects strongly indicates that oxidative stress is a major player in DOX-induced bone loss [71]. Skeletal deformities, altered meristic characters, and delayed development can be considered developmental disturbances that indicate inappropriate nutrition and associated oxidative stress [12,13]. Malondialdehyde (MDA) concentration is the hallmark for lipid peroxidation resulting from oxidative stress by exogeneous production of ROS by food containing high polyunsaturated fatty acid, ionizing radiation aging, and environmental factors [55,120]. This study shows that dietary supplementation with antioxidants is beneficial to overcome the oxidative stress resulting from external dietary sources. Our results show that the inclusion of antioxidants in larval diets for *S. aurata* can help prevent skeletal problems during early development, with benefits for the production of this species.

In the present study, supplementation with antioxidants resulted in improved protection against DOX-induced peroxidation, as evidenced by the significant reduction of MDA concentration. This demonstrates the importance of RES and MT to reduce oxidative stress by reducing hydroperoxides [55,120]. Rana et al. reported that SOD1 is a molecular target of DOX-mediated bone loss [72]. In the present study, the phenotype induced by DOX in both in vitro and in vivo experiments has been reversed by performing a co-treatment with antioxidants. We hypothesized that RES-induced reversal of DOX-induced bone deformities in seabream larvae is related to increased osteoblast differentiation/mineralization and protection against oxidative stress, as indicated by our in vitro studies. DOX has previously been shown to reduce bone mass in humans and mice [48,49,50] as a result of decreased osteoblastogenesis [72] or increased osteoclastogenesis [121]. Oxidative stress is one of the major players in DOX-induced bone loss, in agreement with data previously published in which SOD1 was identified as a molecular target of DOX-mediated bone loss [72]. Our in vitro study shows that the decrease in the mRNA of *sod1* was significantly reversed by RES. Transcriptomic analysis has revealed p53 as a key regulator of doxorubicin-induced toxicity in mice [122]. In addition, p53 was shown to induce expression of pro-oxidants genes to further increase ROS, resulting in apoptosis and senescence of the cells [123]. In contrast, RES was shown to increase osteoblastogenesis by inhibiting the p53 signaling pathway on human bone marrow-derived mesenchymal stem cells [124]. Altogether, the reversal of the negative effects of DOX upon bone cell differentiation and mineralization, and the increase in expression of antioxidant genes by RES, suggests that p53 is a major player involved in the mechanism of DOX-induced bone loss. Further studies should be carried out to confirm the proposed role of p53 on the mechanism of bone differentiation. 

## 5. Conclusions

In conclusion, our data indicate that DOX-induced mineralization, bone deformities, and bone loss are a result of oxidative stress. Antioxidant supplementation effectively prevented the incidence of bone anomalies and mineralization defects induced by pro-oxidants in both in vivo and in vitro models. RES and MT supplementation were able to reverse pro-oxidant-induced effects on bone anomalies, mineralization, and oxidative stress. Further testing in other models will reinforce our findings. Our data further suggest that antioxidants supplementation of fish diet would be beneficial to overcome oxidative stress-induced bone deformities. 

## Figures and Tables

**Table 1:** Ingredient’s formulation in the experimental microdiets.

Ingredients (g/kg)	Control	Resveratrol	MitoTEMPO	Doxorubicin
Squid powder	702.00	702.00	702.00	702.00
Gelatin	30.00	30.00	30.00	30.00
Krill oil	130	130	130	130
Mineral premix	45.00	45.00	45.00	45.00
SelPlex	3.00	3.00	3.00	3.00
Vitamin premix	60.00	60.00	60.00	60.00
Attractants	30.00	30.00	30.00	30.00
Resveratrol	---	0.034	---	---
MitoTEMPO	---	---	0.005	---
Doxorubicin	---	---	---	0.030

**Table 2:** Vitamins, minerals, and attractants formulation in the experimental microdiets.

Vitamin Premix		Mineral Premix	g/kg
Hydro-soluble Vitamin	g/kg	NaCl	2.15133
Cyanocobalamin B12	0.0003	MgSO4.7H2O	6.77545
Astaxanthin	0.05	NaH2PO4.H2O	3.81453
Folate	0.0544	K2HPO4	7.58949
Pyridoxine B6	0.1728	Ca(H2PO4).2H2O	6.7161
Thiamine B1	0.2177	FeC6H5O7	1.46884
Riboflavin B2	0.7253	C3H5O3.1/2Ca	16.1721
Pantothenata calcium B5	1.0159	Al2(SO4)3.6H2O	0.00693
4-Aminobenzoic acid	1.45	ZnSO4.7H2O	0.14837
Nicotinic acid B3	2.9016	CuSO4.5H2O	0.01247
Inositol	14.509	MnSO4.H2O	0.02998
Sub Total	21.097	KI	0.00742
Lipo-soluble Vitamin	g/kg	CoSO4.7H2O	0.10706
Retinoic acid Vit A	0.0024	Total	45.00007
Cholecalciferol Vit D3	0.0365		
Menadione Vit K	0.1728	Attractants	g/kg
α-Tocopherol acetate (Vit E Acetate)	1.5	Inosine-5-monophosphate (Inosinc acid)	5
Sub total	1.7117	Betaine (Trimethylglycine)	6.6
Ascorbyl polyphosphate (V.C)	1.8	L-Serine	1.7
Choline Chloride	29.658	L-Tyrosine	1.7
		L-Phenylalanine	2.5
Vitamin premix Total	54.2667	DL-Alanine	5
		L-Aspartic acid	3.3
		L-Valine	2.5
		Glycine	1.7
		Total	30

**Table 3:** List of considered anomalies.

Regions affected
A. Cephalic (first–second vertebra; carrying epipleural ribs)
B. Pre-haemal or Pleural (With open haemal arches carrying epipleural or pleural ribs, without haemal spines)
C. Haemal or pre caudal (With haemal and neural arches closed by spines)
D. Caudal (With haemal and neural arches closed by modified spines)
E. Pectoral fin
F. Anal fin
G. Caudal fin
H. Dorsal fin
**Anomalies Considered**
1. Skoliosis
2. Lordosis
3. Kyphosis
4. Vertebral fusion
5. Vertebral body malformation
6. Malformed neural arch and/or spine
7. Malformed haemal arch and/or spine and/or rib
8. Malformed ray (deformed, absent, fused, supernumerary)
9. Malformed pterygiophores (deformed, absent, fused, supernumerary)
10. Malformed hypural (deformed, absent, fused, supernumerary)
11. Malformed epural (deformed, absent, fused, supernumerary)
12. Others
13. Jaw deformities
14. Reduced dental/malformed Pre-maxillary and/or maxillary
15. Cephalic deformities (glossohyal, neurocranium, etc.)
16. Vertebral slipping
17. Deformed or reduced opercle
18. Supernumerary vertebra
19. Ectopic mineralization
20. Absent

**Table 4:** Primer sequences.

Gene	GenBank (Accession No.)	5′–3′ Primer Sequence
*catalase (cat)*	Q308823.1	Fw: TGGTCGAGAACTTGAAGGCTGTC
		Rev: AGGACGCAGAAATGGCAGAGG
*glutathione peroxidase 1 (gpx1)*	KC201352.1	Fw: GAAGGTGGATGTGAATGGAAAAGATG
		Rev: CTGACGGGACTCCAAATGATGG
*superoxide dismutase 1 (sod1)*	XM_030439011.1	Fw: TGACGCTCACAGGAGAAATCAAAGGG
		Rev: CAGTAGGACCGCCATGATTCTTACCA
*heat shock protein 90 kDA alpha 1 (hsp90)*	KM522802.1	Fw: TGCCTGGAACTCTTCACCGAACTG
		Rev: CGCAGCAGATCAGACAACTTCTTCCT
*osteopontin (spp1)*	AY651247.1	Fw: TACCATCGTCACGGACACAGAGACAG
		Rev: GCTCGTAGGACTTGTAGGGAACAGG
*ß-actin (actb)*	AF384096.1	Fw: CTTCCTCGGTATGGAGTCCTGCGG
		Rev: TCCTGCTTGCTGATCCACATCTGCT

**Table 5:** Meristic character count.

		Cranial Vertebrae		Pre-Haemal Vertebrae		Haemal Vertebrae		Caudal Fin				DORSAL FIN			Anal Fin		
		Mineralized	Mineralizing	Mineralized	Mineralizing	Mineralized	Mineralizing	Epural	Ectopic C. in hypuralia	Rays (Mineralizing)	Rays (Unmineralized)	Pterygiophores (Cartilaginous)	Lepidotrichia (Mineralizing)	Lepidotrichia (Unmineralized)	Pterygiophores (Cartilaginous)	Lepidotrichia (Mineralizing)	Lepidotrichia (Unmineralized)
CONTROL	Mean	0.3667 *	0.2367 *	2.0633 *	1.28 *	0.0533 *^δ^	0.15 *	3.53 *	0.7567 *	13.78 *	2.58 *	11.92 *	0.87 *	6.4533 *	7.6967 *	0.8133 *^δ^	5.1933 *^δ^
	Std.	0.4827	0.42575	3.08074	1.55014	0.37987	0.56737	0.67623	0.81	3.24552	2.18774	9.08085	2.87064	6.88422	5.24624	2.72947	5.36955
RES	Mean	0.5304 *	0.1791 *	3.1993 *	1.3311 *	0.1824 *	0.1723 *	3.4561 *	0.6723 *	14.6723 *	2.0676 *	12.0946 *	1.4899 *	5.8277 *	7.9122 *	1.375 *	4.5946 *
	Std.	0.49992	0.38405	3.42238	1.37724	0.88327	0.62222	0.58056	0.78	2.98085	1.96981	8.98272	3.50785	5.91413	5.01784	3.08379	4.88975
MT	Mean	0.4047 *	0.1271 *^δ^	2.4314 *	0.9699 *^δ^	0.1271 *	0.1271 *	3.5017 *	0.5585 *	12.903 *	2.9431 *	10.5786 *	0.9264 *^δ^	5.9331 *	6.6656 *	0.8361 *^δ^	4.5786 *
	Std.	0.49165	0.33363	3.27762	1.38148	0.75345	0.53461	0.51406	0.708826785	3.11318	2.1253	9.51913	2.79524	6.15479	5.50246	2.52967	4.88076
DOX	Mean	0.4392 *	0.125 *^δ^	2.3547 *	0.8851 *	0.0541 *^δ^	0.0507 *	3.4662 *^δ^	0.5946 *^δ^	13.9189 *	2.2399 *	13.5878 *	1.2568 *	8.2061 *	8.3649 *^δ^	1.0878 *	6.1723 *
	Std.	0.49713	0.33128	3.14279	1.3405	0.46989	0.27457	0.63689	0.677361159	2.97564	2.04688	9.15251	3.15717	7.18577	5.07055	2.84737	7
DOX+RES	Mean	0.5973 *	0.1107 *	3.4497	0.9564 *^δ^	0.1242 *	0.2483 *	3.4732 *^δ^	0.5906 *^δ^	14.4832 *	2.1745 *	13.1745 *	2.2483 *	6.6946 *	8.3289 *^δ^	1.8658 *	5.151 *
	Std.	0.49126	0.31434	3.49205	1.16115	0.6777	0.68565	0.55135	0.756684446	2.78187	1.59009	8.81324	4.49452	6.41946	4.96173	3.73654	4
DOX+MT	Mean	0.5498 *	0.0859 *	2.9416 *	1.055 *	0.0893 *	0.1649 *	3.4777 *	0.7595 *	14.0344 *	2.5636 *	12.2337 *	1.488 *	7.0172 *	7.7629 *	1.2749 *	5.5326 *
	Std.	0.49837	0.28071	3.24622	1.22773	0.62056	0.5933	0.52063	0.781602051	3.05109	1.68929	9.20871	3.71702	6.71358	5.29822	3.03429	5.36285

^δ^ No significant differences between the group, * Significant differences between the groups (one-way ANOVA, Tukey’s multiple comparisons test, *p* ≤ 0.05). Acronyms: Std.: Standard deviation, resveratrol (RES), doxorubicin (DOX), MitoTEMPO (MT), doxorubicin + resveratrol (DOX+RES), doxorubicin + MitoTEMPO (DOX+MT), and Ectopic C. in hypuralia (Ectopic cartilage in hypuralia).

## Supplementary Materials

The following supporting information can be downloaded at: https://www.mdpi.com/article/10.3390/nu14061154/s1, Figure S1: Survival of seabream larvae. Survival of seabream on waterborne exposure on different concentrations of DOX for 48 h (A), survival of the larvae up to 24 h with microdiets prepared (B); Figure S2: Mineral analysis of gilthead seabream fed with microdiets. Sodium (A), potassium (B), magnesium (C), and iron (D). One-way ANOVA, Tukey’s multiple comparisons test, ns—*p* > 0.05, *—*p* ≤ 0.05, **—*p* ≤ 0.01, ***—*p* ≤ 0.001, and ****—*p* ≤ 0.0001; Table S1: Temperature, Oxygen, and O_2_ Saturation during the seabream trial.
