# Supplementary material for: Reversal of Doxorubicin-Induced Bone Loss and Mineralization by Supplementation of Resveratrol and MitoTEMPO in the Early Development of Sparus aurata
Source: Nutrients. 2022 Mar 9;14(6):1154. doi: 10.3390/nu14061154 (PMC8950850; doi:10.3390/nu14061154)
Supplement: Supplementary file 1 [file nutrients-14-01154-s001.zip › nutrients-1611626-supplementary.pdf]

Supplementary Materials:

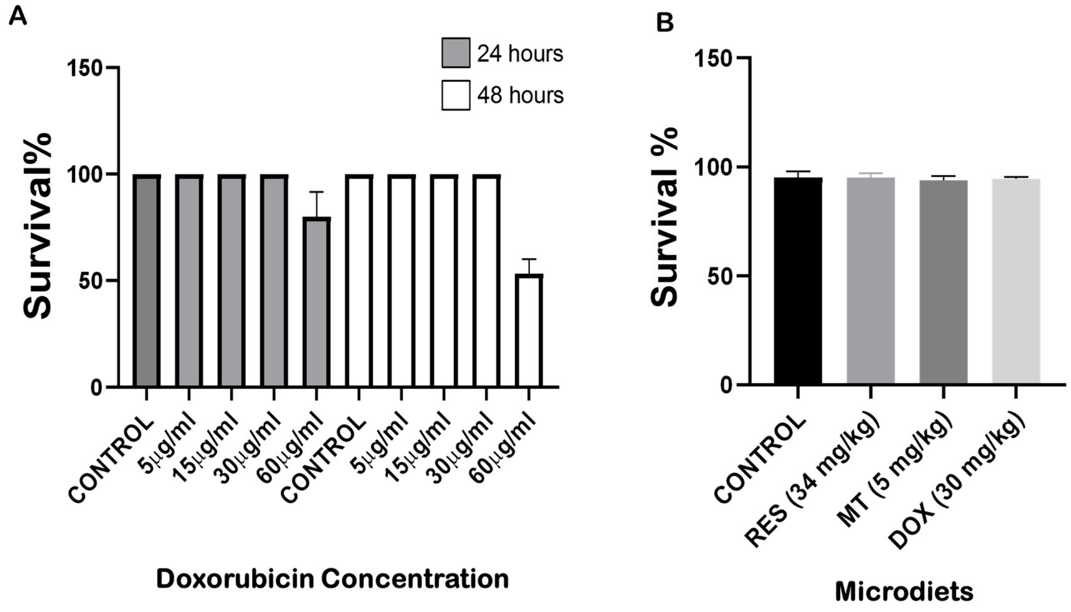

Figure S1. Survival of gilthead seabream larvae. Survival of seabream on waterborne exposure on different concentration of DOX for 48 hours (A), survival of the larvae up to 24 hours with microdiets prepared (B). Graph prepared in Graphpad prism 8 ±SEM. Acronyms: Resveratrol (RES), Doxorubicin (DOX) and MitoTEMPO (MT).

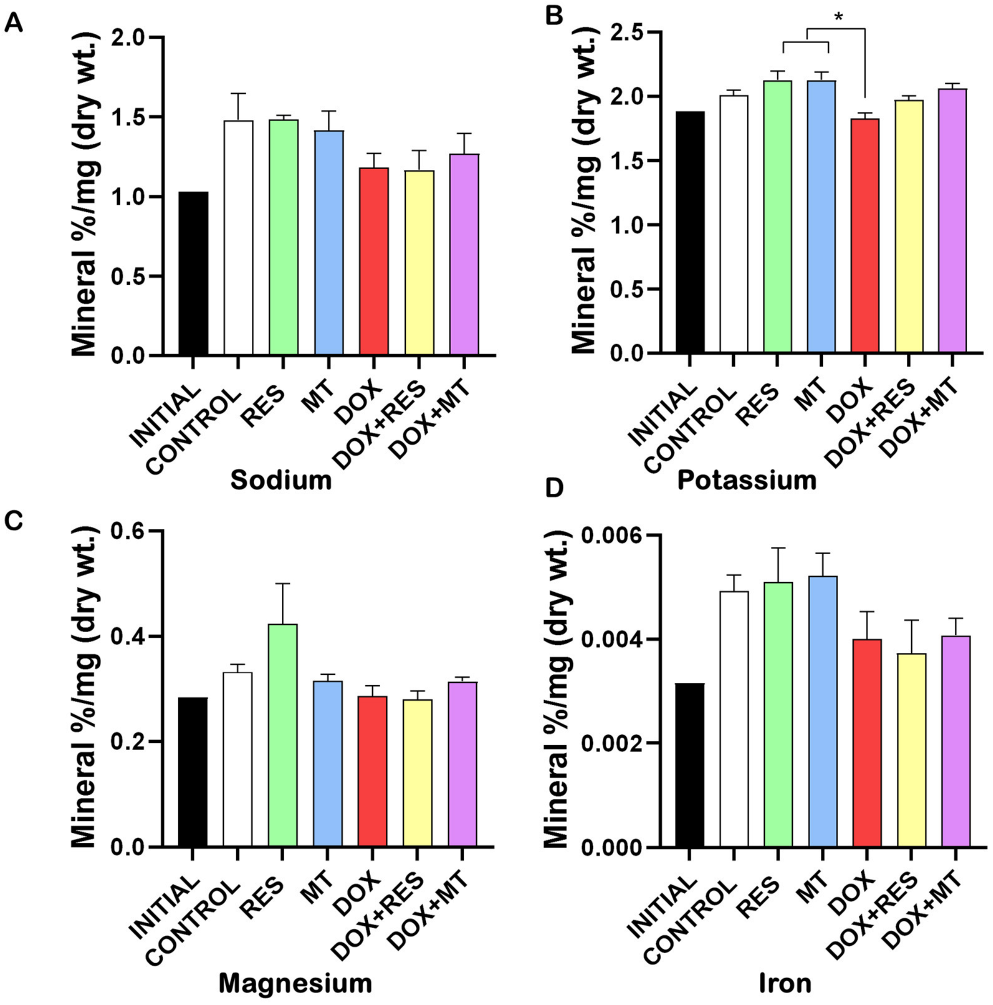

Figure S2. Mineral analysis of gilthead seabream fed with antioxidants and pro-oxidant supplemented microdiets. Sodium (A), potassium (B), magnesium (C) and iron (D). One-way ANOVA, Tukey's multiple comparisons test, *- $P \leq 0.05$. Acronyms: Resveratrol (RES), Doxorubicin (DOX), MitoTEMPO (MT), Doxorubicin+Resveratrol (DOX+RES) and Doxorubicin+MitoTEMPO (DOX+MT).

Table S1. Temperature, oxygen, and oxygen saturation during the seabream rearing trial.

Temperature (°C)	CONTROL	RES	MT	DOX	DOX+ RES	DOX+MT
Mean	20.18	20.16	20.08	20.06	20.06	20.07
Std. Deviation	0.253	0.2606	0.2025	0.1819	0.2288	0.2739
Std. Error of Mean	0.08001	0.08241	0.06403	0.05753	0.07236	0.08661
Oxygen (mg/l)						
Mean	6.557	6.31	6.387	6.55	6.533	6.53
Std. Deviation	0.1325	0.2644	0.1679	0.123	0.1324	0.1401
Std. Error of Mean	0.04188	0.0836	0.0531	0.03889	0.04187	0.04429
Saturation (%)						
Mean	89.5	84.93	86.57	89.07	88.87	88.57
Std. Deviation	1.408	4.277	2.577	1.858	1.906	2.031
Std. Error of Mean	0.4451	1.352	0.8151	0.5875	0.6029	0.6422

Acronyms: Resveratrol (RES), Doxorubicin (DOX), MitoTEMPO (MT), Doxorubicin+Resveratrol (DOX+RES) and Doxorubicin+MitoTEMPO (DOX+MT).
